# Supplemental Blue LED Lighting Array to Improve the Signal Quality in Hyperspectral Imaging of Plants

**DOI:** 10.3390/s150612834

**Published:** 2015-06-01

**Authors:** Anne-Katrin Mahlein, Simon Hammersley, Erich-Christian Oerke, Heinz-Wilhelm Dehne, Heiner Goldbach, Bruce Grieve

**Affiliations:** 1 Institute of Crop Science and Resource Conservation (INRES)-Phytomedicine, University of Bonn, Meckenheimer Allee 166a, 53115 Bonn, Germany; E-Mails: ec-oerke@uni-bonn.de (E.O.); hw-dehne@uni-bonn.de (H.D.); 2 School of Electrical and Electronic Engineering, University of Manchester, Oxford Road, Manchester M13 9PL, UK; E-Mails: simon.hammersley@manchester.ac.uk (S.H.); bruce.grieve@manchester.ac.uk (B.G.); 3 Institute of Crop Science and Resource Conservation (INRES)-Plant Nutrition, University of Bonn, Karlrobert-Kreiten-Strasse 13, 53115 Bonn, Germany; E-Mail: h.goldbach@uni-bonn.de

**Keywords:** hyperspectral imaging, plant reflectance, blue LED array, signal-to-noise

## Abstract

Hyperspectral imaging systems used in plant science or agriculture often have suboptimal signal-to-noise ratio in the blue region (400–500 nm) of the electromagnetic spectrum. Typically there are two principal reasons for this effect, the low sensitivity of the imaging sensor and the low amount of light available from the illuminating source. In plant science, the blue region contains relevant information about the physiology and the health status of a plant. We report on the improvement in sensitivity of a hyperspectral imaging system in the blue region of the spectrum by using supplemental illumination provided by an array of high brightness light emitting diodes (LEDs) with an emission peak at 470 nm.

## 1. Introduction 

Hyperspectral imaging is commonly used to study the characteristics of plants in a certain environment, or their reaction to abiotic (drought, nutrient deficits, heavy metals) or biotic (plant diseases, pests, weeds) stress at different scales, from remote to proximal sensing [1,2]. The visible spectrum (VIS, 400–700 nm) is mainly influenced by the absorbance of leaf pigments (chlorophylls, carotenoids, xanthophylls, and anthocyanins) [3,4], and many studies focus on the use of visible reflectance in plant science. However the reflectance of light at blue wavelengths (400–500 nm) is often neglected due to technical limitations, despite relevant information on the optical properties of plants, such as absorbance maxima of chlorophyll a, chlorophyll b and ß-carotene being contained in this region of the electromagnetic spectrum. Changes in the composition of these pigments are a first indicator of plant stress and can be assessed by hyperspectral imaging [5] and, as a consequence, several vegetation indices, based on reflectance wavelength around 450 nm, have been introduced. For example, the Normalized Phaeophytinization Index (NPQI = (R_415_ − R_435_)/(R_415_ + R_435_)) [6] has been applied as an early detection of spider mites in apples. The Plant Stress Index (PSR = R_430_/R_680_) or the Structure Independent Pigment Index (SIPI = (R_800_ − R_435_)/(R_800_ + R_680_)) have been developed to evaluate the ratio among carotenoids and chlorophylls [6]. Mahlein *et al.* [7] found a high relevance of the blue reflection in correlation to the disease severity of powdery mildew and rust in sugar beet. However, the technical performance of different hyperspectral imaging or non-imaging sensors in the blue region is often limited by the noise *versus* the available signal. The spectral response of a hyperspectral system depends upon two principal factors, firstly, the spectral sensitivity of the hyperspectral camera sensor and, secondly, the spectral profile of the illuminating source, which is often a black body emitter [8]. Unfortunately, in the region between 400 and 500 nm, both the sensitivity of hyperspectral camera sensors and the amount of available light delivered by the illuminating source are typically low resulting in a poor signal-to-noise ratio for spectral signatures in this range. Nevertheless, these hyperspectral systems with halogen bulbs as a stable and diffuse light source are well established and proven equipment in manifold studies assessing plant properties non-invasively [9,10,11,12]. Therefore the objective of the present work was to improve the sensor signal by the addition of additional illumination in the blue region. A supplemental blue light-emitting diode (LED) lighting array with an emission peak at 470 nm was developed and tested under controlled conditions. 

## 2. Experimental Section 

### 2.1. Hyperspectral Measuring Setup

**Figure 1:**
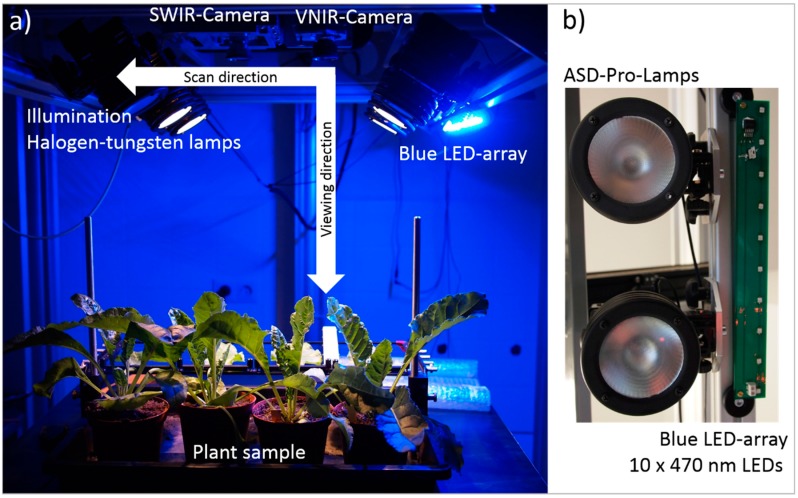
(**a**) Hyperspectral imaging setup for measurements of the reflectance of plants under controlled conditions with artificial illumination; and (**b**) installation of the supplemental Blue LED-array next to the ASD-Pro-Lamps. (Best viewed in colour).

Hyperspectral images were recorded with a line scanning spectrograph (ImSpector V10E, Spectral Imaging Ltd., Oulu, Finland) covering the VIS and the NIR ranges from 400 to 1000 nm, with a spectral resolution of up to 2.8 nm and a spatial resolution of 0.12 mm per pixel, resulting in 210 hyperspectral bands (Figure 1a). As experimental plants sugar beets, cultivar Pauletta (KWS, Einbeck, Germany), diseased with *Cercospora* leaf spot and sugar beet rust were used (plant material was prepared according to [9]). Constant illumination was provided by six ASD-Pro-Lamps (Analytical Spectral Devices Inc., Boulder, CO, USA) (Figure 1b). The hyperspectral camera and the illumination system were installed on a motorized line scanner (Spectral Imaging Ltd.) to obtain a second spatial dimension. The camera settings and the control of the motorized line scanner were adapted using the SpectralCube software (Version 3.62, 2000, Spectral Imaging Ltd.). Hyperspectral images were recorded in a dark chamber in order to realize constant and reproducible illumination and measurement conditions. Normalization of raw hyperspectral images was performed using the software ENVI 4.6+IDL 7.0 (EXELIS Visual Information Solutions, Boulder, CO, USA). Reflectance was calculated relative to a white reference bar and to dark current measurement. 

### 2.2. Supplemental Blue LED Lighting Array

The supplemental illumination board was configured as an array of 10 × 470 nm LEDs (Avago ASMT-JL31, Avago Technologies, San José, CA, USA). This array was mounted in parallel, next to the six ASD-Pro-Lamps of the hyperspectral system. The sample of interest was then illuminated with a constant intensity (Figure 1a, b). This was achieved by spacing the LEDs on a printed circuit board (PCB) in such a manner that the resulting intensity of illumination along the camera’s scan-line was uniform at the sample surface. The PCB also accommodated the constant current source for the LEDs (ON Semiconductor—CAT4101), screw terminals for power connections and mounting points for connecting it to the motorized scanning stage.

## 3. Results and Discussion

The applicability of a simple supplemental blue LED lighting array with an emission peak at 470 nm was developed and tested under controlled conditions for hyperspectral imaging of sugar beet leaves. 

### 3.1. Noise Profile

**Figure 2:**
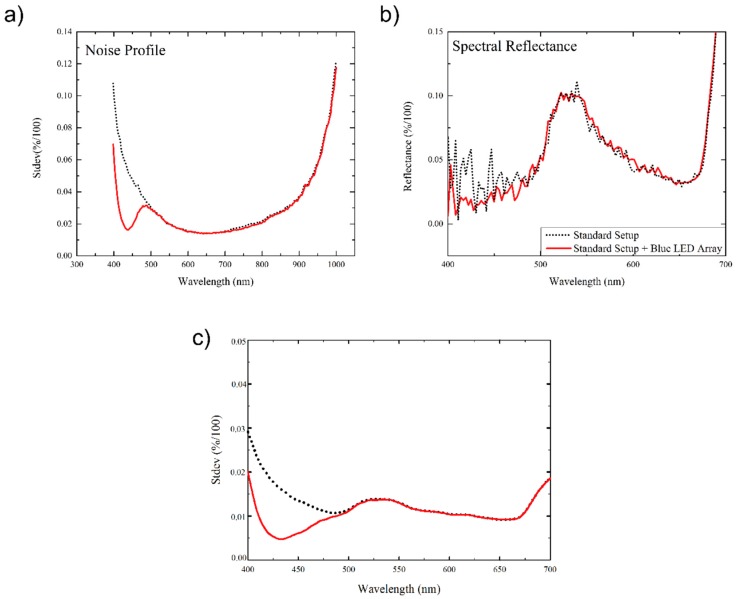
(**a**) Noise profile of the hyperspectral measurement system in reflectance wavebands from 400–1000 nm (the black dotted line indicate the standard setup, the red line the standard setup with the additional blue LED-array); (**b**) spectral reflectance profile of healthy sugar beet leaf tissue in the VIS from 400–700 nm and (**c**) standard deviation of the reflectance of 15,000 pixel of healthy sugar beet leaves without (dotted black line) and with (solid red line) additional illumination by the LED-array. (Best viewed in colour).

To measure the noise profile of the hyperspectral system, the hyperspectral image profile of a white, barium sulphate, reference bar was recorded. The standard deviation of the measured reflectance values, as a function of wavelength, was then computed. With reference to the dotted black line in Figure 2a, the measured noise profile for the original ASD-Pro-Lamp illuminated system, without supplemental LED lighting, shows a high standard deviation in measured values at both extremes of the spectral range, *i.e.*, in the 400–500 nm visible (blue colour) and 900–1000 nm near infrared regions. In these areas the measured standard deviation may represent greater than 10% of the measured signal for the white reference bar. 

The noise profile for the system with the inclusion of the supplemental illumination, as depicted by the solid red line of Figure 2a, indicates an appreciable reduction in the standard deviation in the range 400–500 nm. Figure 2b illustrates a comparison of the reflectance spectrum recorded for the same region of a healthy sugar beet leaf for the system with and without supplemental LED lighting array. The data without the use of the supplemental lighting array shows significantly greater noise in the 400–500 nm region of the spectrum *versus* the equivalent readings with the dual lighting configuration. This effect could potentially be improved still further with the use of a data pre-processing step, such as the application of a Savitzky-Golay filter [13]. Figure 2c visualizes the standard deviation of the reflectance data measured across the same region (15,000 pixels in area) of the surface of a healthy sugar beet leaf. A noticeable reduction in the standard deviation is observed in the blue region (400–500 nm) and can be as much as 75% at 430 nm. 

**Figure 3:**
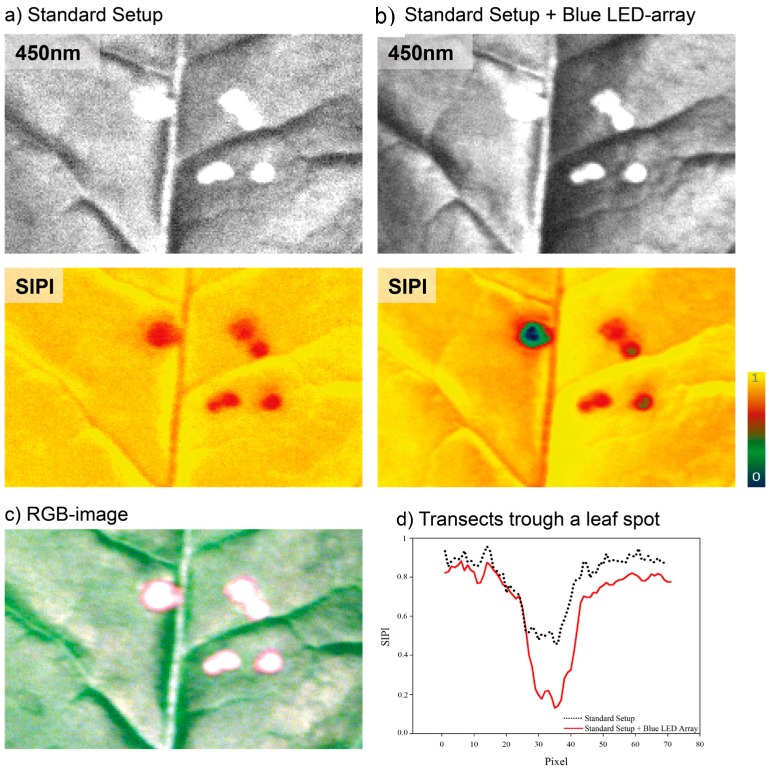
(**a**) Greyscale image of a region of sugar beet leaf diseased with *Cercospora* leaf spot, at 450 nm; (**b**) false colour images of the Structure Independent Pigment Index (SIPI) without and with the supplemental LED illumination; and (**c**) a digital red, green and blue (RGB) image of the sugar beet leaf. The greyscale and the SIPI image with additional illumination show apparently less noise, by this single structures are more distinctive and recognizable. This is further emphasized by transects through a leaf spot (indicated by dashed lines) (**d**), were SIPI values measured with the additional LED illumination source provides a more detailed and severable information of the diseased tissue. (Best viewed in colour).

### 3.2. Improved Sensitivity of Spectral Vegetation Indices

The use of the supplemental illumination results in a significant reduction in noise in the images, potentially enabling structures within the leaf surface to be more easily characterized or identified at an earlier stage. Figure 3a,b, show greyscale images of the reflectance of a sugar beet leaf at 450 nm, false colour SIPI images with and without the use of the supplemental LED illumination, Figure 3c an RGB image of the sample and Figure 3d a transect of the SIPI through one of the leaf spots, respectively. Transects trough a Cercospora leaf spot reveal a higher sensitivity and detail rich information using the supplemental LED illumination. As a consequence of further developments in commercial LED and imaging detector components this research indicates that in combination they will enable the delivery of cost-effective hyperspectral sensors systems, for plant science and agriculture that provide greater spectral and spatial sensitivity than it is currently achievable [14].

## 4. Conclusions

This study indicates that the inclusion of a supplemental 400–500 nm (blue light) LED lighting array can significantly improve the sensitivity of hyperspectral plant images, under controlled conditions. The standard deviation of the measured data from a white barium sulphate reference object in this region was observed to decrease by a factor of 4. Tests of this system on healthy and diseased sugar beet leaves showed that both the recorded reflectance spectra and the recorded images in the 400–500 nm region of the spectrum show significantly less signal noise and a significantly lower standard deviation with the proposed additional illumination. The inexpensive and minor—but effective—modification to a hyperspectral imagining system could be applied in future agronomic research to improve the study of abiotic and biotic plant stress symptoms. There are indications that especially wavelength from 400–500 nm are sensitive to early reactions of plants to plant diseases, even before visible symptoms appear. Furthermore the use of the blue LED illumination system will provide more reliable results if vegetation indices with wavelength from 400–500 nm are calculated. This approach can be transferred to different imaging and non-imaging system and is not limited to the camera setup presented. Such modified hyperspectral imaging systems may assist with the detection and identification of plant stress in phenotyping studies. 
